# Co-occurrence of Glycogen Storage Disease Type 2 and Congenital Myasthenic Syndrome Type 5 in a Pediatric Patient: A Case Report

**DOI:** 10.7759/cureus.26345

**Published:** 2022-06-26

**Authors:** Fawzia Al-Sharif, Mohammed F Alamer, Hussein O Taher, Raneem Y Gazzaz, Asma O AlRuwaithi, Tuleen T Miliany, Mohammed A Alrufaihi, Abdullah F Al Amer

**Affiliations:** 1 Pediatrics, Saudi German Hospital Jeddah, Jeddah, SAU; 2 College of Medicine, Batterjee Medical College, Jeddah, SAU; 3 College of Medicine, Ibn Sina National College, Jeddah, SAU; 4 College of Medicine, Gulf Medical University, Ajman, ARE

**Keywords:** autosomal recessive, autoimmune, infant, enzyme replacement therapy, congenital myasthenia syndrome type 5, glycogen storage disease type 2

## Abstract

Glycogen storage disease type 2 (also known as Pompe disease) is a metabolic disorder characterized by an accumulation of glycogen within lysosomes. Pathophysiologically, this condition is caused by an autosomal recessive deficiency of the lysosomal acid alpha-glucosidase enzyme, resulting in defects in lysosomal metabolism. Glycogen accumulation causes advanced muscle weakness (myopathy) throughout the body, including the heart, skeletal muscles, liver, and the neurological system. Currently, there is no definitive treatment for Pompe disease. However, recent studies have indicated that enzyme replacement therapy (ERT) can be effective. Myasthenia gravis (MG) is an autoimmune illness that affects the postsynaptic acetylcholine receptors and causes fatigue that can be eased by rest. MG is frequently accompanied by a thymoma.

Dyspnea and/or bulbar symptoms can indicate an imminent crisis requiring immediate intervention. Here, we present a rare case of a four-year-old female patient who initially presented at the age of one month with the infantile form of Pompe disease and congenital myasthenia syndrome type 5. The patient presented with bradycardia, poor suckling, respiratory distress, and respiratory failure requiring assisted ventilation, subglottic stenosis, and tachypnea. Whole exome sequencing was used for definitive diagnosis. ERT (Myozyme) was administered with good results. We propose that early identification and management of Pompe disease with Myozyme can improve patients’ condition and ultimately increase the possibility of survival.

## Introduction

Glycogen storage disease (GSD) type 2, also known as Pompe disease or acid maltase deficiency, is an autosomal recessive disease characterized by impairments in the glycogenesis and glycogenolysis pathways. Multiple gene mutations that can trigger deficiencies of the lysosomal enzyme acid alpha-glucosidase (GAA) have been identified as potential causes of GAA deficiency. The global incidence of Pompe disease is reported to be 1:40,000. According to a study performed in Saudi Arabia over 25 years (1983-2008), of the 165,530 Saudi infants investigated, three were diagnosed with GSD type 2 (Pompe disease), indicating an incidence rate of one in 100,000 live births [1]. This condition can be further classified according to the organ involved, age of onset, rate of progression, and severity. The most common clinical manifestations include substantial weakness of skeletal muscles, including both the motor and respiratory muscles.

Myasthenia gravis (MG) is an autoimmune illness that affects the postsynaptic acetylcholine receptors and causes fatigue that can be eased by rest. MG is frequently accompanied by a thymoma. Approximately 2-3% of MG patients are admitted with crisis each year, necessitating intubation and ventilation. MG can be classified into two major forms: acquired autoimmune and congenital form [2]. Congenital myasthenia syndrome is an inherited disorder in which the safety margins of neuromuscular transmission are impaired by one or more specific mechanisms and is characterized by muscle weakness that is worsened upon exertion. It comprises several phenotypically and genetically heterogeneous disorders that are familial and lack autoantibodies against the acetylcholine receptors. More frequent mutations were found in the choline acetyltransferase (ChAT), Collagen like tail subunit of asymmetric acetylcholinesterase (ColQ), and acetylcholine receptor (AChR) subunits [3].

## Case presentation

This four-year-old female child was initially referred to the Saudi German Hospital from East Jeddah Hospital at one month for further management of respiratory distress. She was placed on mechanical ventilation. The patient was delivered by spontaneous vaginal delivery (SVD); her birth weight was 2.99 kg with Appearance, Pulse, Grimace, Activity, and Respiration (APGAR) scores of 5 and 8 at 1 and 5 minutes, respectively. She was connected to a bubble continuous positive airway pressure (CPAP) but showed no improvement, resulting in intubation and connection to a mechanical ventilator. On admission, her weight was 2.810 kg. Several attempts to wean her from mechanical ventilation failed after resulting in respiratory distress with desaturation and bradycardia. Laryngoscopy was performed that revealed weak vocal cord movement with subglottic stenosis and a membrane-like structure under the cords indicating a laryngeal web, which was managed surgically with a recommendation for tracheostomy. After tracheostomy, the baby was gradually weaned from mechanical ventilation but still experienced attacks of desaturation and respiratory distress, sometimes necessitating mechanical ventilation. An oral feeding trial failed because of poor suckling; therefore, an orogastric tube (OGT) was inserted to help feeding. Laryngoscopy and bronchoscopy were later performed, which revealed normal movement of the vocal cords and granuloma related to the laryngeal web membrane and above the tracheostomy tube. Lavage was performed, and the fluid sent for a culture and sensitivity test, revealing methicillin-resistant Staphylococcus aureus (MRSA). The patient was maintained on IV Targocid for seven days on room air with only tracheostomy tube care and frequent chest physiotherapy for every day of the failed oral feeding trial. Feeding was performed by OGT with 50 ml/2 h and was tolerated well; thus, gastrostomy tube feeding was initiated. The patient remained under observation on tracheostomy care, OGT feeding, and gastrostomy tube.

## Discussion

Pompe disease is a metabolic myopathy caused by mutations in the gene for GAA, the enzyme that degrades glycogen into glucose within the acidic milieu of the lysosome [4]. A deficiency of this enzyme causes a lysosomal buildup of glycogen in several tissues [5]. Genetic analyses have revealed that it is caused by mutations of encoding genes in chromosome 17q25.2-q25.3. Variant interpretation of whole-exome sequencing is as follows:

For congenital myasthenia syndrome type 5: COLQ, c.241_242dup p. (Asn81Lysis*19). The COLQ variants create a shift in the reading frame tearing at codon 81. The new reading frame ends in a stop codon 18 positions downstream. This variant was also identified in a heterozygous state in the parents and was classified as likely pathogenic (class 2) according to the recommendation of Centogene (Rostock, Germany), and the American College of Medical Genetics and Genomics (ACMG) pathogenic variants in the COLQ gene are associated with congenital proximal muscle weakness and respiratory insufficiency (Table 1).

**Table 1:** Whole-Exome Sequencing

Patient	Mother	Father
Homozygous pathogenic variant was identified in the GAA gene, confirming the genetic diagnosis of GSD type 2. A homozygous pathogenic variant was further identified in the COLQ gene. Given the autosomal recessive mode of inheritance, a genetic diagnosis of congenital myasthenic syndrome type 5 was also confirmed.	Heterozygote pathogenic variant was identified in the GAA gene, and a heterozygote likely pathogenic variant was identified in the COLQ gene.	Heterozygote pathogenic variant was identified in the GAA gene, and a heterozygote likely pathogenic variant was identified in the COLQ gene. Along with the maternal findings, this confirmed the homozygote of the detected variant in the patient.

For GSD type 2: The GAA c.1828G>A p. (Ala610 Thr) mutation was identified. The GAA variant c.1828G-A p. (Ala610 Thr) causes an amino acid change from Ala to Thr at position 610. This disease usually presents as progressive muscle weakness, reduced muscular tone, cardiomyopathy, and respiratory deficiency within the first three months of life [6]. Our patient presented with bradycardia, poor suckling, respiratory distress, respiratory failure requiring assisted ventilation, subglottic stenosis, and tachypnea. Feeding and swallowing problems in addition to respiratory difficulty, which are often combined with upper and lower respiratory tract infections, are common. Our patient had difficulty feeding and was thus maintained on OGT. The only currently approved therapy for Pompe disease is Myozyme enzyme replacement therapy (ERT). Myozyme, produced by recombinant DNA technology using a Chinese hamster ovary cell line, is administered as an intravenous infusion once every two weeks. It is identical in amino acid sequence to a commonly occurring human form of GAA [7]. Clinical trials to study the safety and effectiveness of ERT in Pompe disease began in 1999 with a transgenic product derived from rabbit milk. Overall, these trials showed that Myozyme provides a considerable benefit when used in Pompe disease, especially when started early in the disease course. ERT (Myozyme) acts by restoring lysosomal GAA activity, resulting in the stabilization and restoration of cardiac and skeletal muscle function. In a previous trial, patients started receiving Myozyme at the age of one year twice a week [7]. Trials were conducted to allow the increased opportunity for potential improvement and survival, with the hope of moving toward maximizing physical function. The patient, who was additionally prescribed long-term physical therapy sessions, showed marked respiratory improvement with improved physical movement.

## Conclusions

Early detection of Pompe disease by whole-exome sequencing can potentially slow disease progression and enhance patients' quality of life; consequently, newborn screening procedures are prospective modalities for improving Pompe disease outcomes. Treatment with enzyme replacement therapy (ERT) can impressively change the course of the disease, thus improving the quality of patients’ lives and decreasing the incidence of the development of critical complications. The key to the successful use of ERT is achieving an early diagnosis of GSD type II. A precise study of the selected categories of patients with myodystrophy and other myopathic syndromes at any age is required to recognize late-onset Pompe disease to begin pathogenetic treatment as early as possible.
